# The contribution of cooking appliances and residential traffic proximity to aerosol personal exposure

**DOI:** 10.1007/s40201-020-00604-7

**Published:** 2021-01-22

**Authors:** M. Shehab, F. D. Pope, J. M. Delgado-Saborit

**Affiliations:** 1 School of Geography, Earth and Environmental Sciences, University of Birmingham, Edgbaston, Birmingham, B15 2TT UK; 2 Environmental Protection Authority (EPA), Shuwaikh Industrial, Kuwait City, Kuwait; 3 Perinatal Epidemiology, Environmental Health and Clinical Research, School of Medicine, Universitat Jaume I, Castellon, Spain; 4 ISGlobal Barcelona Institute for Global Health, Barcelona Biomedical Research Park, Barcelona, Spain

**Keywords:** Personal exposure, Airborne pollutants, Particulate matter (PM_2.5_), Black carbon (BC), Ultrafine particles (UFP), Indoor/outdoor exposure

## Abstract

**Purpose:**

Indoor and outdoor factors affect personal exposure to air pollutants. Type of cooking appliance (i.e. gas, electricity), and residential location related to traffic are such factors. This research aims to investigate the effect of cooking with gas and electric appliances, as an indoor source of aerosols, and residential traffic as outdoor sources, on personal exposures to particulate matter with an aerodynamic diameter lower than 2.5 μm (PM_2.5_), black carbon (BC), and ultrafine particles (UFP).

**Methods:**

Forty subjects were sampled for four consecutive days measuring personal exposures to three aerosol pollutants, namely PM_2.5_, BC, and UFP, which were measured using personal sensors. Subjects were equally distributed into four categories according to the use of gas or electric stoves for cooking, and to residential traffic (i.e. houses located near or away from busy roads).

**Results/conclusion:**

Cooking was identified as an indoor activity affecting exposure to aerosols, with mean concentrations during cooking ranging 24.7–50.0 μg/m^3^ (PM_2.5_), 1.8–4.9 μg/m^3^ (BC), and 1.4 × 10^4^–4.1 × 10^4^ particles/cm^3^ (UFP). This study also suggest that traffic is a dominant source of exposure to BC, since people living near busy roads are exposed to higher BC concentrations than those living further away from traffic. In contrast, the contribution of indoor sources to personal exposure to PM_2.5_ and UFP seems to be greater than from outdoor traffic sources. This is probably related to a combination of the type of building construction and a varying range of activities conducted indoors. It is recommended to ensure a good ventilation during cooking to minimize exposure to cooking aerosols.

**Supplementary Information:**

The online version contains supplementary material available at 10.1007/s40201-020-00604-7.

## Introduction

People who live on busy roads are more likely to suffer adverse health effects [1, 2]. A study by Carey et al. (2016) in London suggested that people living on or close to busy roads may increase the risk of exacerbating health problems related to heart failure and pneumonia at short-term exposure [3]. Living close to traffic roadside has also been related to an increased risk of dementia [4, 5] and cognitive decline [6–8], slower rate of cognitive development [9], structural changes in the brain [10, 11], neurotoxicity [12] and neurobehavioural problems in children such as autism spectrum disorders [13]. Recently, short term exposure to PM_2.5_ has been linked to short term cognitive decline [14]. Evidence also suggests a contribution of exposure to air pollution to the risk of developing cardiometabolic syndrome [15]. Indoor sources might also contribute to ill health. Jarvis et al., (1996) mentioned that people who use gas stoves, as opposed to electric, at home experience more respiratory-related health problems [16]. Likewise, other studies have also found associations between exposure to indoor air pollution and respiratory health effects [17–24]. Indoor exposures, mainly associated with tobacco smoke, have also been related to increased risk of lung cancer [25]. Moreover, epidemiological evidence suggests that the associations between adverse health effects and black carbon (BC) exposure (a carbonaceous component of particulate matter emitted during incomplete combustion) are stronger than for PM_2.5_ [26, 27].

Outdoor air and residential traffic are important contributors to exposure to air pollution. In addition, there are multiple indoor sources that contribute to air pollution exposure [28]. Cooking is an important source contributing to indoor air and personal exposure [29]. A study by He et al. (2004) found that indoor ultrafine particle (UFP) (particles with an aerodynamic diameter of 100 nm or less) concentrations can be elevated by up to 5 times due to activities related to cooking, including frying, grilling, stove use, toasting. Other activities contributing to indoor sources include fan heaters and candles [30]. Particulate matter with aerodynamic diameter less than 2.5 μm (PM_2.5_) concentrations can be higher than background levels by up to 3, 30, and 90 times due to smoking, frying and grilling respectively [30]. Géhin et al. (2008) found the highest emissions concentrations when cooking meat or fish whether in stove or in oven [31]. Other cooking related activities also affect the PM_2.5_ concentrations at home, including baking, broiling, basting and roasting, which can affect human health and can lead to morbidity and mortality [32].

Since people spend the majority of their time in indoors at home, it is expected that indoor sources, including cooking, pet dander, environmental tobacco smoke (ETS), burning of candles and incense sticks, as well as the use of household cleaning agents would contribute to the exposure to various components of particulate matter, such as UFP, PM_2.5_ and BC [32]. This is in addition to pollutants that originate from outdoor sources, which penetrate or infiltrate into the house [33]. Elevated concentrations of air pollutants can remain indoors even after indoor activities have concluded. This is relevant for particles emitted during cooking (which is a major indoor source), ETS, and those from incense stick burning, where the airborne particles from tobacco smoke and incense stick burning remain for longer than particles from cooking [34]. For instance, Hussein et al.’s (2006) study found that fine particles emitted from smoking one cigarette are equal to the amount of particles produced during approximately half an hour of cooking, and that airborne particles from tobacco may remain up to ten hours.

Studies assessing personal exposure to a large range of aerosols metrics concurrently are still scarce. Many of the studies assessing personal exposures have focused on measuring one or two aerosol metrics [35–39], but studies reporting BC, PM_2.5_ and UFP concurrently are very limited [40, 41]. In addition, a comparative assessment of the influence of indoor and outdoor sources on the personal exposure to particulate matter is also very limited [42–46].

This research assesses the effect of indoor and outdoor sources on personal exposure to different aerosol size fractions (UFP and PM_2.5_) and constituents (BC) during time spent in a residential indoor microenvironment (i.e. the home), including active and sleeping times. The main indoor source considered is cooking with different types of appliances (gas compared with electricity). The outdoor source considered is residential traffic (i.e. living near a busy road). This work presents valuable information related to aerosol exposures for epidemiological studies.

## Methods

### Subject’s recruitment and related information

The criteria for the recruitment of subjects was that they were healthy, non-smoking, non-occupationally exposed adults. Pregnant and nursing women were excluded to take part in the study in compliance with EPA’s regulations regarding protection of vulnerable groups in 40 Code of Federal Regulation Part 26. Details of the recruitment process can be found in the supporting information and in Delgado-Saborit et al. (2018) [47].

Forty subjects were recruited (24 females and 16 males), and grouped in four categories according to two criteria: Residential traffic exposure, i.e. location of home with reference to traffic (traffic roadside/ non-traffic roadside), and type of cooking appliance stove hob (Gas/Electricity). Location of homes on A & B roads were selected as traffic roadsides homes. Ten subjects were assigned to each group as summarized in Table S1 (Supplementary Material).

Sampling was conducted in Birmingham (UK) from 6 December 2014 to 25 March 2016. Each subject was sampled for 24 h, for four consecutive days.

Each subject was given a folder including a set of forms to be filled during their sampling. The forms were designed based on previous studies [48, 49] and are available at Delgado-Saborit et al. (2018) [47]. The forms collected information on all activities done by the subject (Activity Diary); recorded and described all locations visited outdoors or in transit (Location sheet for in transit locations); and indoors (Location sheet for static locations); provided information about smoking if subjects had been exposed to second hand smoke (ETS questionnaire); as well as describing activities that may have affected or produced pollutants (Sampling questionnaire). The detailed list of forms and instructions given to the subjects can be found in the Supplementary Information.

### Instruments and equipment

Personal exposure (PE) of particulate matter (PM_2.5_), black carbon (BC), and ultrafine particles (UFP) were collected for forty subjects by using MicroPEM™ v 2.7 personal monitor for PM_2.5_, MicroAeth™ model AE51 personal monitor for BC, and portable sensor Testo DiSCmini for UFP.

The MicroPEM™ measures PM_2.5_ particles in real time using a nephelometric optical bench. In addition, it collects particles downstream the nephelometer using an integrated Teflon filter (25 mm) allowing for gravimetric measurement. The MicroAeth™ model AE51 personal monitor provides real time BC analysis by measuring the rate of change in absorption of transmitted light due to continuous collection of air sample deposits on a Teflon coated glass fiber filter strip. The Testo DISCmini sensor detects UFP based on electrical charging of the aerosols. It measures particle sizes ranging from 10 to approximately 700 nm, and measures UFP counts with a diameter below 300 nm.

Detailed information on the three sensors is provided in the supplementary material. Subjects were given a voice recorder to record their daily activities, microenvironments visited and times thus facilitating filling the forms.

### Sampling and data collection

All sensors used were already validated prior to personal exposure sampling (Delgado-Saborit et al., 2018) [47]. Measurements were collected with time resolution according to each sensor: for the MicroAeth™ which measures BC, a 5-min time interval; for the microPEM which measures PM_2.5_, 10 s; and for the DiSCmini sensor which measures UFP, a 1 s time interval. The timescales were then integrated to time intervals of 5 min (for PM_2.5_ and UFP), 1 h, and 24 h for all pollutants and data was post-processed to correct for any voltage changes, or flow variations as described in detail in the supporting information.

### Data analysis

Minitab statistical software version 17.1.0 was used to test the normality of the BC, PM_2.5_ and UFP. According to the normality results, non-parametric Mann-Whitney tests were applied to conduct a comparative analysis of personal exposures (5% significance level) to test a) whether personal exposure while cooking with a gas stove is higher than cooking with an electrical stove; and b) whether personal exposure to pollutants while spending time in houses located near busy roads is higher than time at houses located near quiet roads.

To compare the emission of aerosols during cooking according to cooking source, the following datasets were compared:

- A. Personal exposure in houses using gas stoves (G) compared to houses using electric stoves (E) for subjects living in houses located in busy roads (TR).
- B. Personal exposure in houses using gas stoves (G) compared to houses using electric stoves (E) for subjects living in houses located in quiet roads (NTR).

These analyses were conducted in the subset of data representing two specific time frames: times where subject reported to be at home, and times where cooking took place.

To assess the effect of traffic as an indoor source of aerosol, the following datasets were compared:

- C. Personal exposure in houses located near busy roads (TR) compared to houses located in quiet roads (NTR) for subjects living in houses that use gas stove (G) for cooking.
- D. Personal exposure in houses located in busy roads (TR) compared to houses located away from traffic roads (NTR) for subjects living in houses that use electric stove for cooking.

These analyses were conducted in the subset of data representing two specific time frames: times where subject reported to be at home, and times at home where no indoor activity is likely to emit aerosols, i.e. sleeping time.

## Results

### Statistical and descriptive results

Statistical analysis for normality indicates all the results from BC, PM_2.5_, and UFP are not normally distributed, hence non-parametric Mann-Whitney (M-W) tests were applied to investigate differences between groups.

**Fig. 1:**
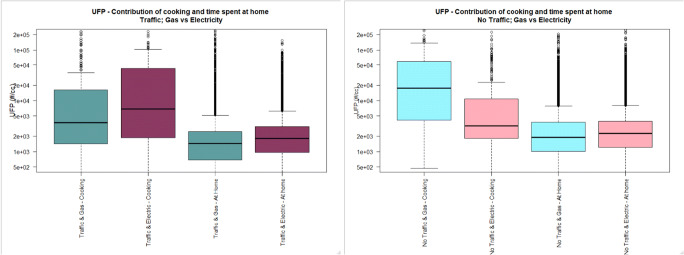
UFP personal exposure concentrations (5-min time average) during cooking, and time spent at home, in houses located either near (dark colour) or away from busy roads (light colour), using either gas (cadet blue/light blue) or electric (dark pink/light pink) stove. The pollutant measurement distributions are non-normal (see main text) and axis are truncated. Full axis boxplots can be found in Fig. S1

**Fig. 2:**
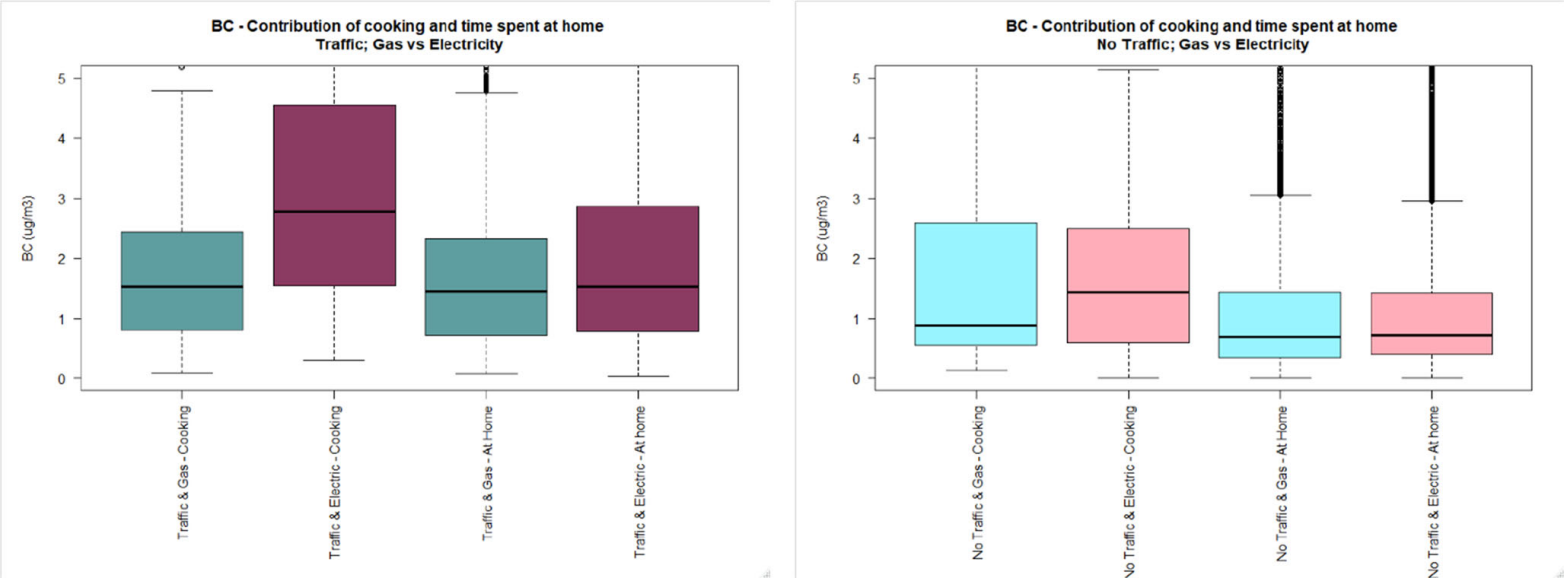
BC personal exposure concentrations (5-min time average) during cooking, and time spent at home, in houses located either near (dark colour) or away from busy roads (light colour), using either gas (cadet blue/light blue) or electric (dark pink/light pink) stove. The pollutant measurement distributions are non-normal (see main text) and axis are truncated. Full axis boxplots can be found in Fig. S2

**Fig. 3:**
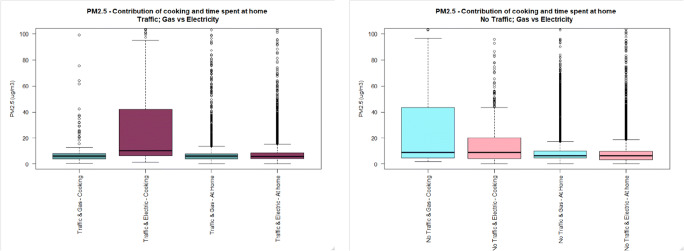
PM_2.5_ personal exposure concentrations (5-min time average) during cooking, and time spent at home, in houses located either near (dark colour) or away from busy roads (light colour), using either gas (cadet blue/light blue) or electric (dark pink/light pink) stove. The pollutant measurement distributions are non-normal (see main text) and axis are truncated. Full axis boxplots can be found in Fig. S3

**Fig. 4:**
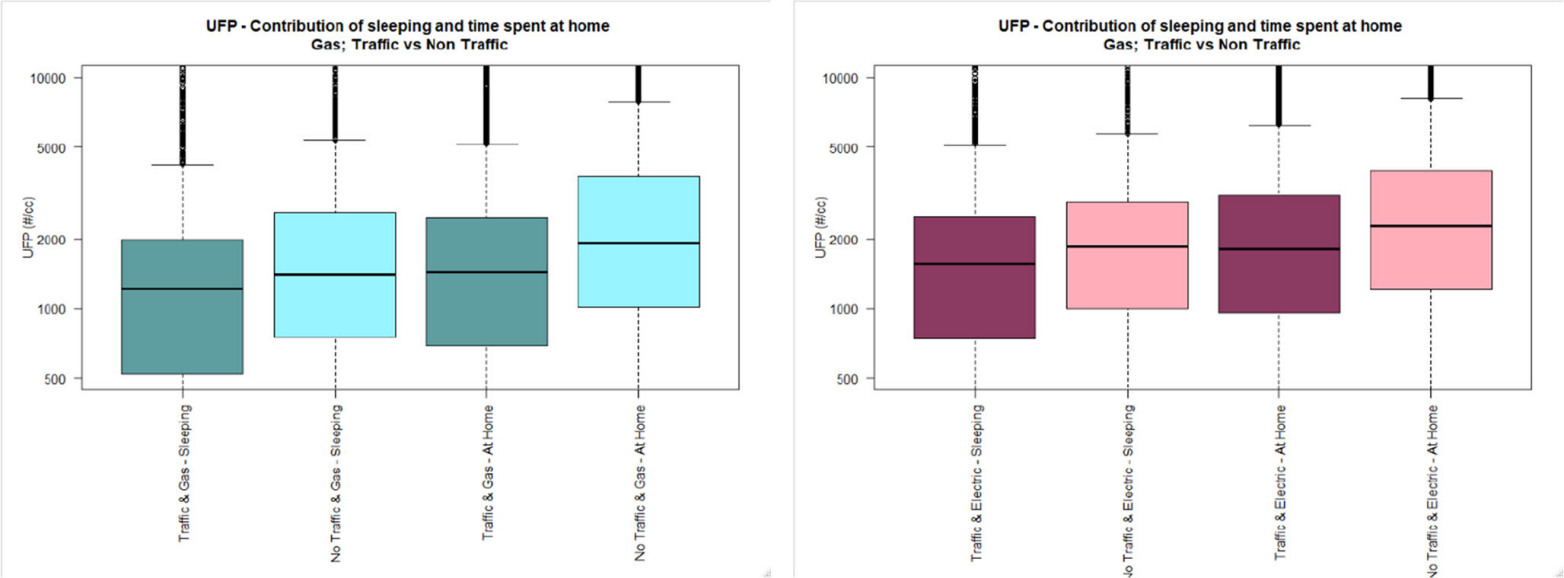
UFP personal exposure concentrations (5-min time average) during sleeping, and time spent at home, in houses located either near (dark colour) or away (light colour) from busy roads, using either gas (cadet blue/light blue) or electric (dark pink/light pink) stove. The pollutant measurement distributions are non-normal (see main text) and axis are truncated. Full axis boxplots can be found in Fig. S4

**Fig. 5:**
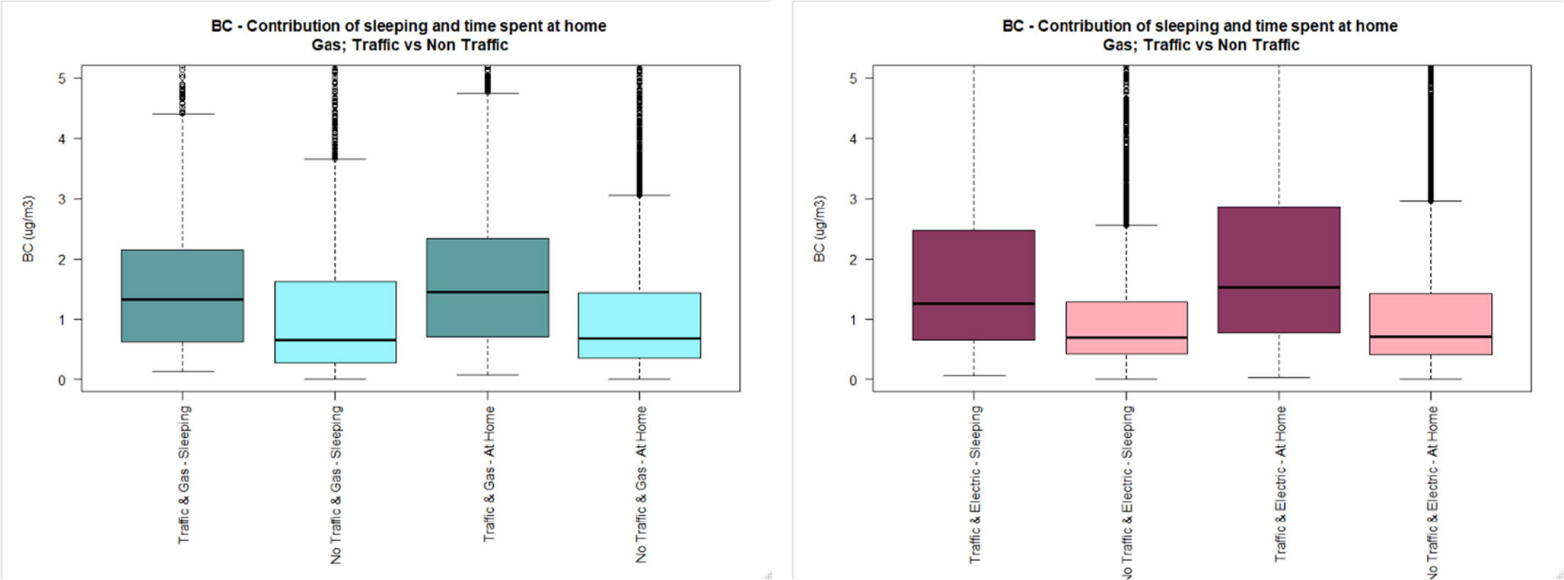
BC personal exposure concentrations (5-min time average) during sleeping, and time spent at home, in houses located either near (dark colour) or away (light colour) from busy roads, using either gas (cadet blue/light blue) or electric (dark pink/light pink) stove. The pollutant measurement distributions are non-normal (see main text) and axis are truncated. Full axis boxplots can be found in Fig. S5

**Fig. 6:**
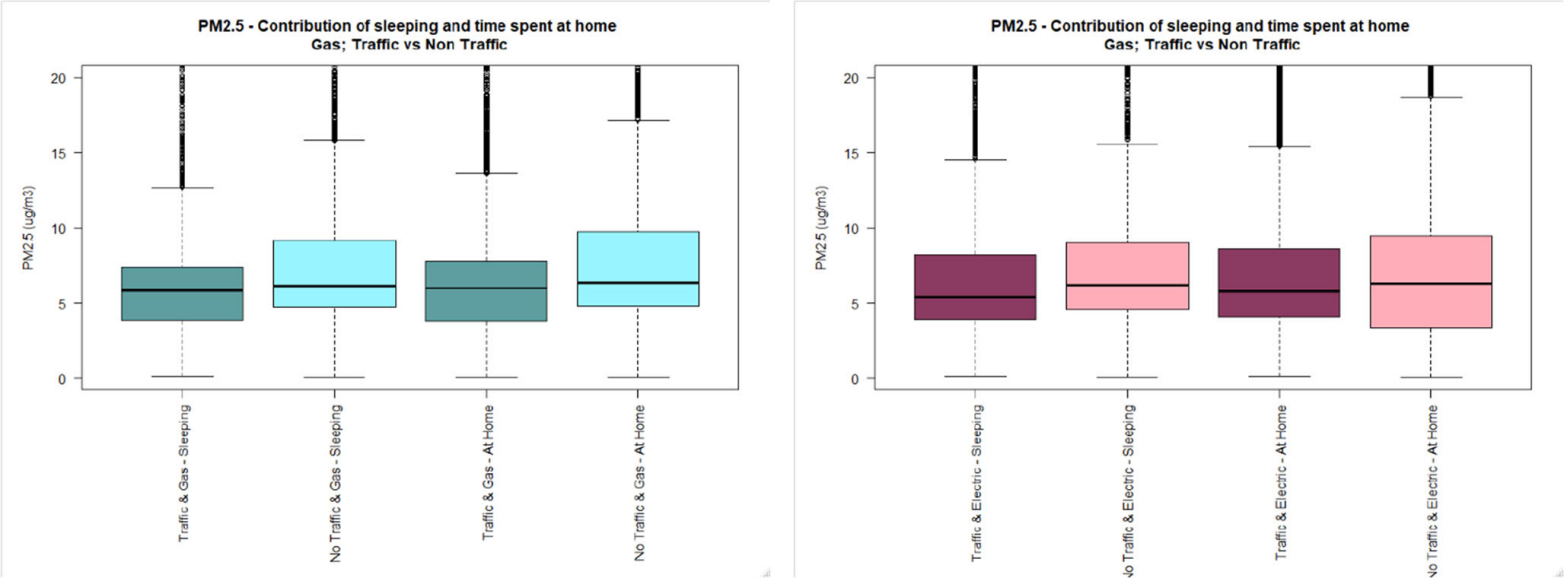
PM_2.5_ personal exposure concentrations (5-min time average) during sleeping, and time spent at home, in houses located either near (dark colour) or away (light colour) from busy roads, using either gas (cadet blue/light blue) or electric (dark pink/light pink) stove. The pollutant measurement distributions are non-normal (see main text) and axis are truncated. Full axis boxplots can be found in Fig. S6

The general characteristics of the study population can be found in Table 1. Only one subject was exposed to environmental tobacco smoke (ETS) during the sampling campaign, and this subject had only one exposure to ETS during this period. Figures 1, 2 and 3 illustrate the distribution of UFP, BC and PM_2.5_ personal exposure for those subjects cooking with gas or electric hobs (Figs. S1-S3 display personal exposure distribution with full-scale axis). Likewise, Figs. 4, 5 and 6 illustrate the distribution of UFP, BC and PM_2.5_ personal exposure for those subjects living in houses located near or away from residential traffic (Figs. S4-S6 display personal exposure distribution with full-scale axis). Table 2 summarizes the results for each pollutant, from the key determinants and activities.

**Table 1:** Characteristics of subjects participating in the study

Characteristic Description		Number of cases	%
Gender	Male	16	40
	Female	24	60
Age range	18–25	17	42.5
	26–35	14	35
	36–45	4	10
	46–55	1	2.5
	56–65	3	7.5
	>66	1	2.5
Ethnicity	White	22	55
	Asian	10	25
	Black	6	15
	Other ethnicities	2	5
Occupation	Student	23	57.5
	Researcher	5	12.5
	Office worker	6	15
	Retired	4	10
	Others	2	5
Tobacco user	Yes	0	0
	No	40	100
ETS Exposure at home	Yes	4	10
	No	36	90
ETS Exposure at workplace	Yes	3	7.5
	No	35	87.5
	N/A	2	5

**Table 2:** Contribution of cooking, time spent at home, and sleeping in houses located either near (TR) or away from busy roads (NTR), using either gas (G) or electric stove (E), on personal exposure, at 5-min time interval

Group	Activity	Pollutant	Key determinant	Median	Mann-Whitney test *p value*/	Number of observations^(a)^
G vs E – TR	Cooking	BC (μg/m^3^)	G	1.5	0.000	464
			E	2.8		341
		PM_2.5_ (μg/m^3^)	G	6.1	0.000	435
			E	10.2		325
		UFP (particles/cm^3^)	G	3674.1	0.0005	256
			E	6829.8		195
	Time spent at home	BC (μg/m^3^)	G	1.4	0.000	8376
			E	1.5		7942
		PM_2.5_ (μg/m^3^)	G	6.0	0.0571	7805
			E	5.8		7526
		UFP (particles/cm^3^)	G	1445.3	0.0000	4675
			E	1801.7		3545
G vs E – NTR	Cooking	BC (μg/m^3^)	G	0.9	0.0028	377
			E	1.4		658
		PM_2.5_ (μg/m^3^)	G	8.7	0.0019	367
			E	8.8		594
		UFP (particles/cm^3^)	G	17,439	0.0000	232
			E	3184		370
	Time spent at home	BC (μg/m^3^)	G	0.7	0.0327	6886
			E	0.7		8142
		PM_2.5_ (μg/m^3^)	G	6.4	0.0000	6389
			E	6.3		7052
		UFP (particles/cm^3^)	G	1904.9	0.0000	4431
			E	2283.2		4596
TR vs NTR – G	Sleeping	BC (μg/m^3^)	TR	1.3	0.000	5174
			NTR	0.6		4374
		PM_2.5_ (μg/m^3^)	TR	5.8	0.0000	4874
			NTR	6.1		4097
		UFP (particles/cm^3^)	TR	1209.5	0.0000	2790
			NTR	1407.6		2672
	Time spent at home	BC (μg/m^3^)	TR	1.4	0.000	8376
			NTR	0.7		6886
		PM_2.5_ (μg/m^3^)	TR	6.0	0.0000	7805
			NTR	6.4		6389
		UFP (particles/cm^3^)	TR	1445.3	0.0000	4675
			NTR	1904.9		4431
TR vs NTR – E	Sleeping	BC (μg/m^3^)	TR	1.2	0.000	5236
			NTR	0.7		5011
		PM_2.5_ (μg/m^3^)	TR	5.4	0.0000	5026
			NTR	6.2		4315
		UFP (particles/cm^3^)	TR	1558.6	0.0000	2448
			NTR	1854.4		2357
	Time spent at home	BC (μg/m^3^)	TR	1.5	0.000	7942
			NTR	0.7		8142
		PM_2.5_ (μg/m^3^)	TR	5.8	0.0000	7526
			NTR	6.3		7052
		UFP (particles/cm^3^)	TR	1801.7	0.0000	3545
			NTR	2283.2		4596

(a) N: number of 5-min measurements

#### Personal exposure during cooking

Personal exposure to BC during cooking was slightly higher for those subjects using electric stove than using gas stove (mean, standard deviation, G-TR: 3.1 μg/m^3^, 8.3), (E-TR: 4.9 μg/m^3^, 7.7), (G-NTR: 1.8 μg/m^3^, 2.3), (E-NTR: 2.3 μg/m^3^, 3.2). This result was irrespective of the location of the house near or far from traffic.

In houses located near busy roads, no difference was observed for mean personal exposure to PM_2.5_ during cooking using electric and using gas stoves (*p* value: 0.587), but the median is marginally higher for those subjects using electric stoves (*P* value: 0.000). However, in houses located away from busy roads, the mean personal exposure to PM_2.5_ during cooking is higher for those subjects using gas stove (50.0 μg/m^3^, 130) than using electric stove (24.7 μg/m^3^, 64.4).

Personal exposure to UFP during cooking in houses located near busy roads is similar to personal exposure to UFP in houses using gas or electric stoves (*p* value: 0.101), but the median is higher for subjects cooking using electric stove than using gas stove (*p* value: 0.0005). However, in houses located away from busy roads, the mean for personal exposure during cooking using gas stove (40,711 particles/cm^3^, 54,776), is higher than using electric stove (14,812 particles/cm^3^, 29,121).

#### Personal exposure during time spent at home

Personal exposure to BC during time spent at houses located near busy roads using electric stove (2.9 μg/m^3^, 14.9) was statistically higher (*p* value <0.05) than using gas stove (1.9 μg/m^3^, 2.5). However, in houses located away from busy roads, no difference was observed in PE between houses fitted with electric or gas stoves (p value: 0.472), but the median PE is higher for those subjects using electric stove than using gas stove (*p* value: 0.0327). Personal exposure during time spent at houses located near busy roads was statistically higher (p value <0.05) than the ones located away from busy roads for both using gas or electric stoves (TR-G:1.9 μg/m^3^, 2.5), (NTR-G: 1.4 μg/m^3^, 3.4), (TR-E: 2.7 μg/m^3^, 14.9), (NTR-E: 1.4 μg/m^3^, 2.2).

Personal exposure to PM_2.5_ during time spent at houses located near busy roads using gas stove (10.6 μg/m^3^, 53.6), is statistically higher (*p* value <0.05) than using electric stove (8.5 μg/m^3^, 14.5). However, in houses located away from busy roads, using electric stove (16.0 μg/m^3^, 101), is higher than using gas stove (13.0 μg/m^3^, 23.3), but the median is slightly higher for using gas stove than using electric stove (*p* value: 0.000). Personal exposure during time spent at houses located away from busy roads is higher than houses located in busy roads in both houses using gas or electric stoves (TR-G: 10.6 μg/m^3^, 53.6), (NTR-G: 13.0 μg/m^3^, 23.3), (TR-E: 8.5 μg/m^3^, 14.5), (NTR-E: 16.0 μg/m^3^, 101).

Personal exposure to UFP during time spent at houses located near busy roads is the same when using gas or electric stoves (*p* value: 0.241), but the median is higher for subjects using electric stove than using gas stove (p value: 0.0000). This is the same for houses located away from busy roads, where personal exposure using gas or electric stove is the same (p value: 0.379), but median is higher for using electric stove (p value: 0.0000). Personal exposure during time spent in houses located away from busy roads is higher than houses located in busy roads, in both houses using gas or electric stoves (TR-G: 4,301 particles/cm^3^, 14,608), (NTR-G: 5,406 particles/cm^3^, 13,758), (TR-E: 4,634 particles/cm^3^, 11,120), (NTR-E: 5,680 particles/cm^3^, 15,814).

#### Personal exposure at home without indoor activities (i.e. sleeping)

Personal exposure to BC whilst sleeping in houses located near busy roads is higher than personal exposure of subjects located away from busy roads independent of the type of appliance used for cooking (TR-G: 1.7 μg/m^3^, 1.8), (NTR-G: 1.4 μg/m^3^, 3.5), (TR-E: 2.5 μg/m^3^, 4.3), (NTR-E: 1.3 μg/m^3^, 2.0).

Personal exposure to PM_2.5_ whilst sleeping in houses located away from busy roads is higher than for those subjects sleeping in houses located near busy roads in houses using electric and gas stoves (TR-G: 7.0 μg/m^3^, 12.0), (NTR-G: 12.2 μg/m^3^, 15.5), (TR-E: 7.5 μg/m^3^, 11.9), (NTR-E: 9.8 μg/m^3^, 30.9).

No difference was observed in personal exposure to UFP whilst sleeping in houses located near or away from busy roads, nor for houses using gas (*p* value: 0.075), or electric stove (p value: 0.470). But the median UFP PE is higher for subjects living in houses located near quiet roads, irrespective of the type of cooking appliance.

## Discussion

This research aims at comparatively assessing the effect of cooking with gas and electric appliances as a source of indoor exposure, and living near busy roads as a source of outdoor pollution contributing to personal exposure. The effect was assessed at three key time periods, including time spent sleeping where no indoor sources are likely present (i.e. sleeping); time spent cooking when the indoor source of interest is active, and overall time spent at home.

### Effect of cooking on personal exposures

The highest personal exposure concentrations experienced by the subjects participating in this study whilst staying indoors were measured when the participants were cooking. The highest increase was observed for concentrations of UFP, raising 2.5 to 15 fold the concentrations measured during time spent at home.

Concentrations of PM_2.5_ measured in this study are within the range of those reported in Australia and Italy [30, 50] and lower than those reported in Singapore and Hong Kong [51, 52]. Concentrations of UFP are similar to those reported cooking dinner in USA [53], cooking with oven and microwave in Australia [30], and cooking in Singapore [51], but lower than concentrations measured in Prague (Czech Republic) [54]. Concentrations of BC are higher than those reported by subjects cooking in an earlier study in Birmingham [48].

Findings from time spent at home are inconsistent with the hypothesis that personal exposure while cooking with a gas stove is higher than cooking with an electrical stove. The results of the present study show that BC concentrations during time spent at home using electric stove are higher than using gas stove. Table 2 also shows that PM_2.5_ concentrations are higher for subjects using electric cooking appliances than subjects using gas appliances in houses located near busy roads. This could be related to the fact that many of the subjects within this category were students living in student hall residences and were exposed to higher concentrations from the student kitchens, which contain several hobs, than otherwise would be experienced in the kitchen of a household of the general population. On the contrary, subjects cooking with gas in houses located near quiet roads experience a larger distribution of PM_2.5_ personal exposure concentrations compared to subjects cooking with electric appliances in houses located near quiet roads. A similar pattern was observed for UFP, with higher concentrations for subjects living in traffic roadsides and cooking with electricity, and higher concentrations for subjects living in homes away from traffic and cooking with gas.

The results suggest that gas and electric appliances give rise to different amounts of indoor pollutants. Electric appliances have been related with higher concentrations of BC during cooking, whereas gas appliances have been associated with higher concentrations of UFP and PM_2.5_ during cooking. In addition to the effect of cooking appliance, and the biased effect caused by participating subjects residing in halls of residence, other factors such as cooking method, and products cooked could affect the results obtained in this study. According to Abdullahi et al. (2013), among the factors affecting cooking aerosol concentration and composition are the combustion process, the type of cooking oil, cooking temperature and style, raw food composition and the splashing incurred by stirring food, which has also been proven to generate considerable amounts of aerosols. UFP and PM_2.5_ can be formed and emitted into the atmosphere through a combustion process which occurs during cooking, and UFP numbers and PM_2.5_ can rise due to cooking fumes containing hot vapors, which subsequently cool and nucleate [29]. However, detailed information on these factors affecting cooking emissions beyond type of cooking appliance was not available in the study and hence could not be considered in the analysis. In addition to cooking factors, other indoor sources such as the use of household cleaning agents, using candles, ETS etc. which can remain indoors for a longer time might have an effect on the PE of subjects indoors.

### Effect of residential traffic on personal exposures

Concentrations of PM_2.5_ and UFP measured in this study are towards the lower range of personal exposures reported in the literature and reviewed by Morawska et al. (2013), which ranged 10.6 to 54 μg/m^3^ for PM_2.5_ and 5.3 × 10^3^ to 3.5 × 10^4^ particles/cm^3^ for UFP [55]. Concentrations of BC are similar to those reported in Birmingham (UK) [48] and Seoul (Korea) [56].

Results from the analysis focused on time spent at home are varied with respect the second hypothesis, i.e. that personal exposure to pollutants while spending time in houses located near busy roads is higher than exposure during time spent at houses located near quiet roads. Only the BC results support the hypothesis, since BC personal exposure is found to be higher during time spent in houses located near busy roads both using gas or electric stoves. But for both PM_2.5_ and UFP, the results show that concentrations were higher for those subjects spending time at home in houses located near quiet roads, with independent of the type of cooking appliance.

A study by Yang et al., 2018 calculated and compared both indoor and outdoor concentrations of PM_2.5_ and found that factors associated with indoor and outdoor air exchange such as meteorological variables, building age, window ventilation and air conditioner use affected the contribution of outdoor aerosol indoors. In their study, indoor PM_2.5_ was seen to come from outdoor sources in the main, although indoor sources also were found to be a noticeable contributory factor [57].

Vu et al. (2017) studied the factors affecting penetration and infiltration of nanoparticles (i.e. UFP) from outdoor origin indoors and found that coagulation and evaporation processes were significant processed contributing to the loss of traffic nanoparticles indoors [58]. The results of Vu’s study are consistent with the current results for UFP showing a smaller contribution from outdoor traffic to personal exposure than from indoor sources.

Likewise, the findings from the analyses focused on the sleeping time support only the hypothesis for BC, where higher BC concentrations were measured for those subjects sleeping in houses located in busy roads, irrespective of the type of cooking appliance used at home. By contrast, PM_2.5_ and UFP concentrations were found to be higher during sleeping time in houses located away from busy roads, irrespective of the type of cooking appliance.

Overall, the results suggest that BC exposures are strongly influenced by residential traffic, since BC is a tracer of diesel exhaust and those subjects residing nearby traffic have higher BC PE than those living in houses located away from busy roads. On the contrary, for the participants of this study, our results suggest that their PM_2.5_ and UFP personal exposures seem to be predominantly affected by pollutants of indoor origin, with a smaller effect on exposures from pollutants that can penetrate and infiltrate inside houses from outdoor sources like traffic. This could be associated to two factors. Firstly, the student halls where most of the participants living in traffic roadsides reside are brand new constructions with building materials that conform to modern standards for energy efficiency; hence are tighter buildings reducing the penetration and infiltration from outdoor air indoors. Secondly, the range of activities conducted in the student rooms where the participants would spent most of their time is limited and these activities are low-emitting sources of aerosols (e.g. studying, watching TV/internet programs, sleeping). On the other hand, most of the participants living in homes away from traffic were residing in older construction building with the full range of indoor activities expected from students, professionals and families with children, and hence a wider variety of low and high emitting sources of aerosols. Therefore, the pattern of indoor sources of airborne pollutants is not comparable in both groups, neither is comparable the penetration and efficiency of outdoor pollutants in both groups. These differential characteristics among groups has likely affected the comparability of the results obtained.

### Study limitations and strengths

One of the main limitations of this study was the difficulty in recruiting a group of participants living in homes located in traffic roadsides comparable to the group of participants living in homes located far away from traffic. As discussed above, the age of the house may have affected the air tightness of the building and the filtration/penetration efficiency of outdoor pollutants indoors. The age of the subjects was also not comparable between groups living in traffic and background houses, influencing the type of activities conducted indoors and their potential as aerosol sources (e.g. more microwave cooking). The different characteristics of the participants in the residential and non residential traffic groups might have had an effect on the results obtained in this study and their comparability with other studies. Therefore, caution is recommended in extrapolating these results to other populations.

This study contributed useful evidence assessing personal exposure to a large range of aerosols metrics concurrently, which is a field of research where evidence is scarce.

## Conclusion

This study has identified cooking as an indoor activity affecting exposure to aerosols, namely PM_2.5_, BC and UFP. Emissions of these aerosols will depend on the type of cooking appliance, as identified in this study, and of other factors reported in the literature summarized by Abdullahi et al. (2013). Therefore, it is recommended to ensure a good ventilation during cooking to minimize exposure to cooking aerosols by using extractor fans or opening doors or windows during cooking.

This study also suggest that traffic is a dominant source of exposure to BC, since the results show that people residing in houses located near busy roads are consistently exposed to higher BC concentrations during time spent at their home than subjects residing in houses away from traffic. In contrast, PM_2.5_ and UFP indoor sources seem to be stronger contributors to personal exposures than outdoor sources related to traffic for the participants of this study, which is likely due to a combination of type of building construction and a varying range of activities conducted indoors. Further research should focus on characterizing the chemical and toxicological properties of aerosols indoors and compare these to the chemical and toxicological signature of outdoor aerosols.

## Supplementary Information

ESM 1 (DOCX 527 kb)

## Data Availability

Data can be made available upon request.
