# Supplementary material for: The contribution of cooking appliances and residential traffic proximity to aerosol personal exposure
Source: J Environ Health Sci Eng. 2021 Jan 22;19(1):307–18. doi: 10.1007/s40201-020-00604-7 (PMC8172705; doi:10.1007/s40201-020-00604-7)
Supplement: Supplementary file 1 — (DOCX 527 kb) [file 40201_2020_604_MOESM1_ESM.docx]

**EFFECT OF INDOOR AND OUTDOOR SOURCES ON EXPOSURES:** **CONTRIBUTION OF COOKING APPLIANCES AND RESIDENTIAL TRAFFIC PROXIMITY TO UFP, BC AND PM_2.5_ PERSONAL EXPOSURE**

**Supplementary Material**

*M. Shehab^1,2^, F. D. Pope^1^, J. M. Delgado-Saborit^1,3,4#*^*

*^1^ School of Geography, Earth and Environmental Sciences, University of Birmingham, Edgbaston, Birmingham, B15 2TT, UK.*

*^2^ Environmental Protection Authority (EPA), Shuwaikh Industrial, Kuwait City, Kuwait*

*^3^ Universitat Jaume I, Perinatal Epidemiology, Environmental Health and Clinical Research, School of Medicine, Castellon, Spain*

*^4^ ISGlobal Barcelona Institute for Global Health, Barcelona Biomedical Research Park, Barcelona, Spain*

** Corresponding author* *delgado@uji.es; delgadjm@bham.ac.uk; juanamaria.delgado@isglobal.org*

1. **Recruitment**

Recruitment was achieved in a number of ways: by sending letters to addresses obtained from databases of volunteers who participated in previous studies, posting an announcement on the my-bham portal website (a University of Birmingham online information hub), announcement leaflets distributed in the university, and by informing colleagues and friends. First, a screening questionnaire was completed by potential volunteers to choose the eligible subjects. Then they read the participant information sheet, and the eligible volunteers who replied after reading the participant information sheet were interviewed to give them information about the study and to explain to them in detail their role in the research, to ask if they have any further questions and to make sure that they understood everything before signing the consent form.

1. **Questionnaires and Forms**

Recruitment forms and leaflets includes announcement leaflet, participant interest letter, announcement posters, online announcement, participant information sheet, screening questionnaire, baseline questionnaire, consent form.

Each subject was given a folder including the following forms during their sampling:

- Confirmation form: signed by Dr. Juana Mari Delgado-Saborit as a project supervisor. This form should always be carried by the subject to confirm that they are carrying scientific instruments and are taking part in a research project.

- Participant instruction sheet: included instructions about the sheets and instruments subjects are carrying

- Activity diary: to record and describe all activities done by the subject

- Location sheet for in transit locations: to record and describe all outdoor locations visited

- Location sheet for static locations: to record and describe all indoor locations visited

- Environmental tobacco smoke (ETS) questionnaire: to give information about smoking if they were exposed to second hand smoke

- Sampling questionnaire: to describe some activities that may affect or produce pollutants

- Withdrawal form: In case participant no longer wanted to proceed with the sampling

- Sensors and charger photos: to show the subject which charger belonged to which sensor

Other documents associated to sampling included:

- Standard operating procedure (SOP), for:

- Subject screening and sampling visits

- Gravimetric determination of filters

- Operating the MicroAeth^TM^

- Operating the MicroPEM

- Operating the Ultrafine particle sensor DiSCmini

- Downloading and checking the data from the ultrafine particle sensor DiSCmini

- Pollutants sampling forms

- Personal and home exposure sampling sheet

- Tyburn central site sampling Sheet for UFP

- Filter weighing chart

All the forms and questionnaires mentioned are available at Delgado-Saborit et al (2017)

1. **Personal sensors**

**Particulate matter (PM_2.5_):** Concentrations of PM_2.5_ were collected at the personal level using the MicroPEM™ v 2.7 monitor, from the RTI International Research Institute. The MicroPEMTM measures PM_2.5_ particles in real time using a nephelometric optical bench. In addition, it collects particles downstream the nephelometer using an integrated Teflon filter (25 mm) allowing for gravimetric measurement. The monitor is lightweight (˂240 g), and small (6.5 × 9.5 × 4 cm), which makes it easy to carry during daily activities, and is also quiet. It operates for up to 48 hours on three AA batteries, and can also run on AC mode connected to the mains. The monitor has a limit of detection of 5 µg/m³ and an operational concentration range of 5-10,000 µg/m³ (RTI International).

**Black carbon:** BC real time concentrations were measured using the MicroAethTM model AE51 personal monitor. The sensor operates for up to 24 hours on a single battery charge, and can be connected to the mains power using an adapter. It provides real time analysis by measuring the rate of change in absorption of transmitted light due to continuous collection of air sample deposits on a Teflon coated glass fiber filter strip (model T60). The measurement range is 0-1 mg/m3 with resolution of 0.001 μg/m3. The measurements time base was set to 300 seconds. This portable personal monitor is small (117 × 66 × 38 mm), and lightweight (280 g) making it easy to carry around during daily activity. It can store 4MB in its internal flash memory, and release the information at a later stage onto the microAethCOM PC software (Air Monitors) to be saved on the local drive.

**Ultrafine particles:** The number of UFP was measured using the portable sensor Testo DiSCmini, which is a suitably small size (9 × 18 × 3.5 cm), with time resolution of up to 1 second (1 Hz). The sensor detects particle sizes ranging from 10 to approximately 700 nm, and measures UFP counts with a diameter below 300 nm, while the concentration ranges from about 1000 to over 1,000,000 particles/m³. The battery lasts up to 8 hours, and the data is recorded on a memory card that can be transferred to an external local drive using a USB cable (Testo company, 2012).

1. **Sampling and Data Collection**

Sensors were located inside a backpack lined on the inside with temperature resistant foam to reduce noise from the sensor pumps. The Tygon sampling tubes were attached to one of the backpack straps at the shoulder area. Power chargers with cables already fitted to the sensors were provided to allow the subjects to directly plug and charge the instruments during their time at the office or at home, minimizing handling of the sensors by the participants.

All sensors were set to be charged overnight in the subject’s bedroom to ensure full charging. Subjects were also requested to plug the sensors to the mains power to recharge upon arrival to the home or to the office following a specific set of printed instructions. Subjects were provided with photos of the sensors and their chargers to indicate which charger belonged to which sensor. Instructions were given to the subjects on the first sampling day and through regular visits during sampling days to ensure everything was going according to specifications.

For the MicroPEM sensor this included checking the flowrate and the battery voltage at the beginning and end of the sampling period. Batteries were replaced when needed. A new pre-weighted filter was placed inside the MicroPEM for each subject. The voltage of the sensor was zeroed prior to the sampling period and any voltage shift was recorded at the end of the sampling period. The integrated Teflon filters from the MicroPEM were weighed in a lab with controlled temperature before and after the sampling. Filters were placed in Petri dishes and labeled with subject ID number. Then filters were conditioned at a constant temperature and relative humidity for 24 hours in the balance room at the lab. An ionizing blower and an α-particle source (210Po) were used to reduce the effect of static electricity on the integrated Teflon filters before weighing them using a Sartorius Model MC5 microbalance.

Preparing MicroAethTM sensors included checking for the flowrate before and after the sampling has been completed, and inserting a new filter strip for each subject. The preparation of the DiscMini sensors involved cleaning the inlets, checking the voltage, and checking the flow rate using an HEPA filter before and after each sampling period.

1. **Data Post-processing**

Upon completion of each sampling week, the data was extracted from all the sensors. The time-series data were plotted in a line chart. Peaks in the data were visually identified and the peak timing was checked against information available on the activity diary sheet. If there was a peak in the data that was not related to information provided in the activity sheet, the subject was requested to recall activities and provide additional information on the particular time where the peak in the data was present.

Before data analysis, concentrations measured by each sensor were post-processed according to the criteria set during the validation of the sensors (Delgado-Saborit et al., 2018). The aim was to reduce the bias in the baseline that occurs from voltage variations, and to correct the negative values measured which occur when the sensor voltage drops.

For UFP, data were corrected using the Fierz equations (Fierz et al., 2008; Fierz et al., 2011) and the validation correction factor intrinsic to each sensor that had been generated during the validation experiments to account for deviations on the instrument flowrate from its set point (Delgado-Saborit et al., 2018).

For BC, Optimized Noise-Reduction Algorithm (ONA) software was used to reduce noise observed in the high frequency collection BC data (Environmental Protection Agency). Afterwards, the data was corrected using the method proposed by Apte and colleagues (Apte et al., 2011) as detailed in Delgado-Saborit et al (2018).

As for PM2.5, some negative measurements were recorded due to the drop in the sensor voltage. To correct this, the baseline was zeroed by adding a number equal to the drop of the baseline. Raising the baseline in all the sampling record determines that all the concentrations would rise accordingly. For the PM2.5 sensors, a correction factor was derived by comparing the concentrations measured by the MicroPEM PM2.5 sensor with the concentration measured by the filter located inside the sensors downstream of the nephelometer (i.e. calculating the gravimetric concentration using the small filter inside the sensor and comparing the gravimetric concentration to the concentration reported by the sensor). The correction factors corresponding to each MicroPEM PM2.5 sensor derived in Delgado-Saborit et al (2018) were applied.

1. **Supplementary Tables**

Table S1: Groups of subjects by key determinant

| Group | Traffic exposure | Cooking gas stove | Number of subjects | Total |
| --- | --- | --- | --- | --- |
| 1 | Yes | Yes | 10 | 40 |
| 2 | Yes | No | 10 |  |
| 3 | No | Yes | 10 |  |
| 4 | No | No | 10 |  |

1. **Supplementary Figures**

| 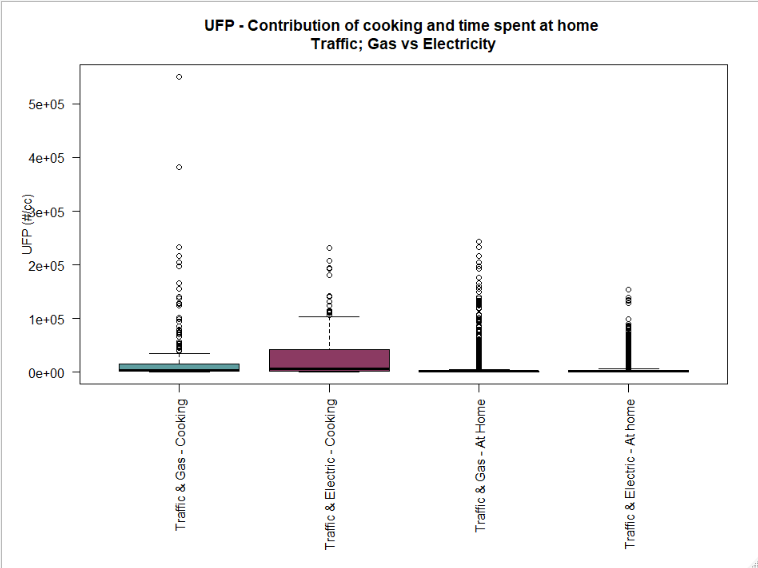 | 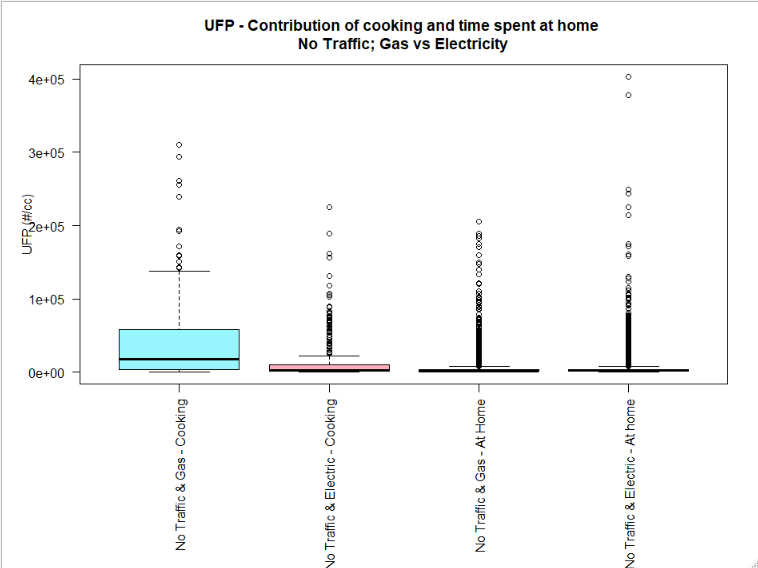 |
| --- | --- |

Figure S1: UFP personal exposure concentrations (5-minute time average) during cooking, and time spent at home, in houses located either near (dark colour) or away from busy roads (light colour), using either gas (cadet blue/light blue) or electric (dark pink/light pink) stove. The pollutant measurement distributions are non-normal (see main text).

| 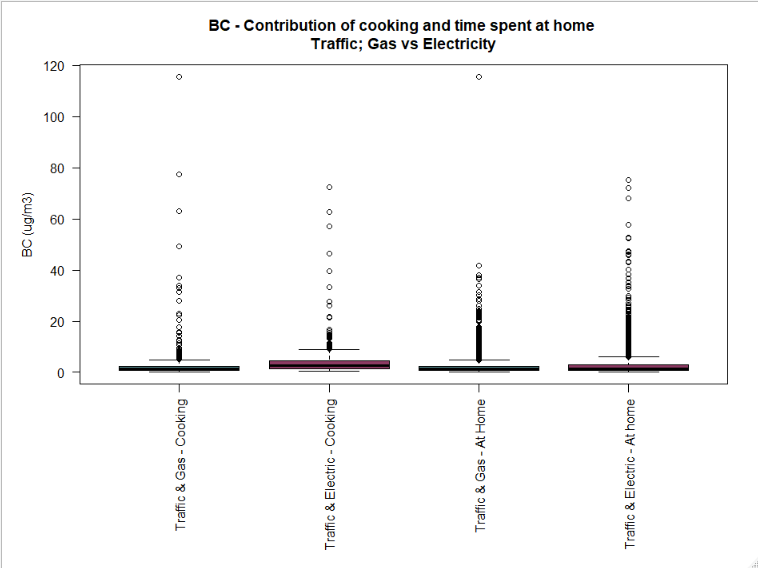 | 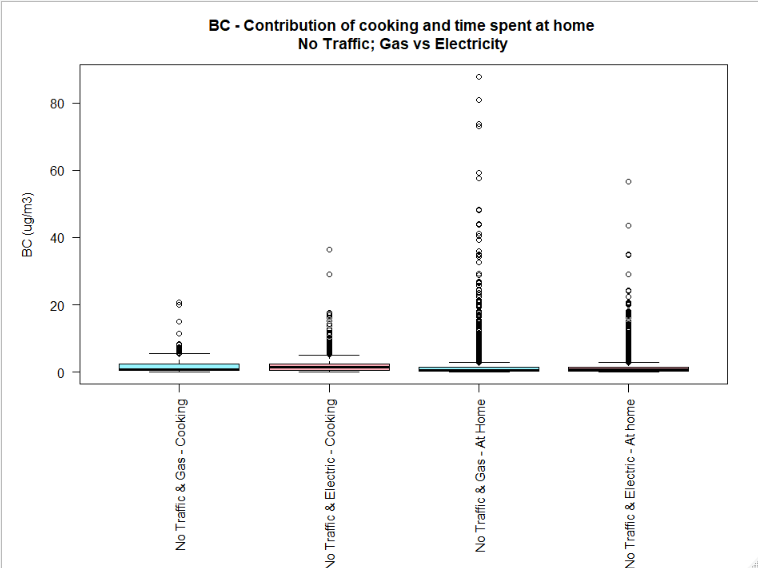 |
| --- | --- |

Figure S2: BC (middle) personal exposure concentrations (5-minute time average) during cooking, and time spent at home, in houses located either near (dark colour) or away from busy roads (light colour), using either gas (cadet blue/light blue) or ele ctric (dark pink/light pink) stove. The pollutant measurement distributions are non-normal (see main text).

| 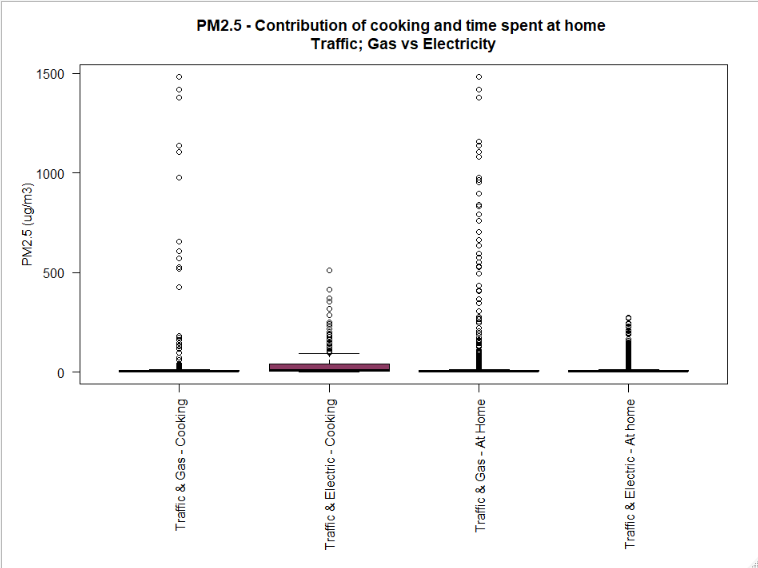 | 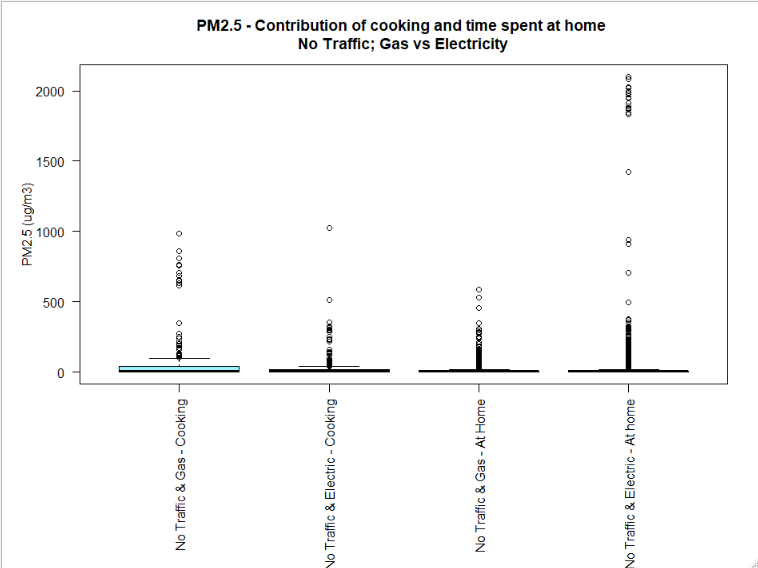 |
| --- | --- |

Figure S3: PM_2.5_ personal exposure concentrations (5-minute time average) during cooking, and time spent at home, in houses located either near (dark colour) or away from busy roads (light colour), using either gas (cadet blue/light blue) or electric (dark pink/light pink) stove. The pollutant measurement distributions are non-normal (see main text).

| 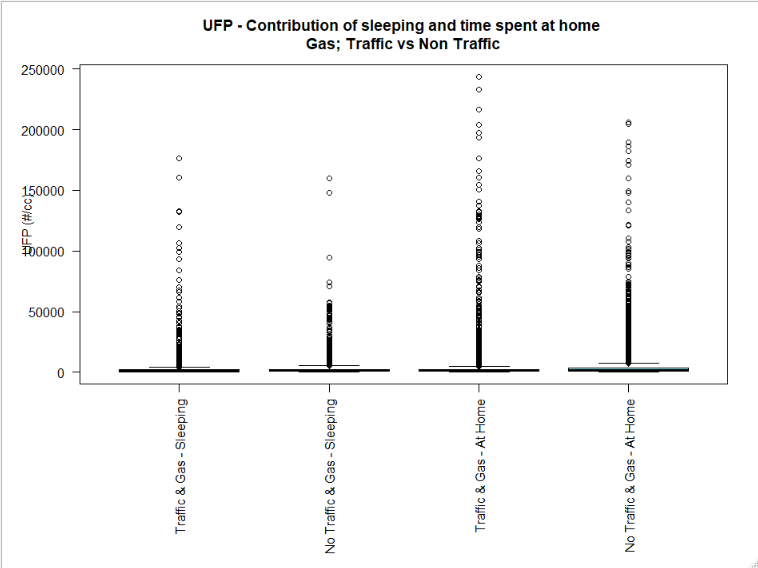 | 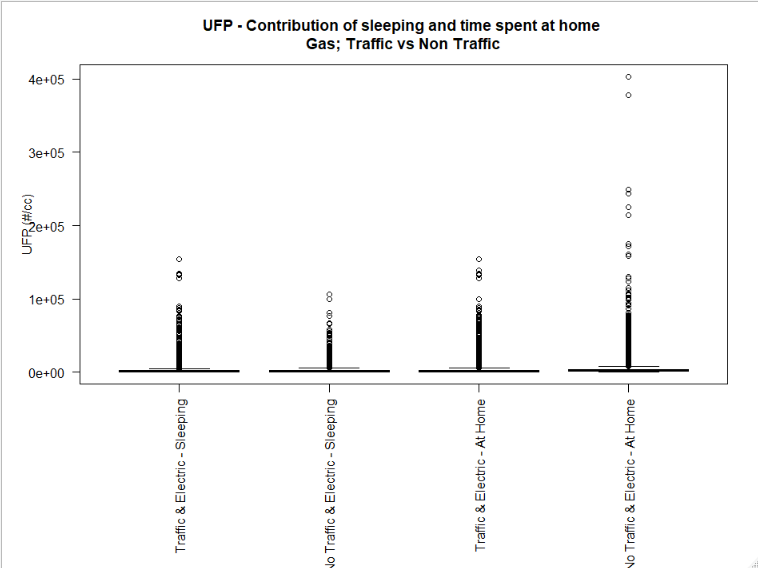 |
| --- | --- |

Figure S4: UFP personal exposure concentrations (5-minute time average) during sleeping, and time spent at home, in houses located either near (dark colour) or away (light colour) from busy roads, using either gas (cadet blue/light blue) or electric (dark pink/light pink) stove. The pollutant measurement distributions are non-normal (see main text).

| 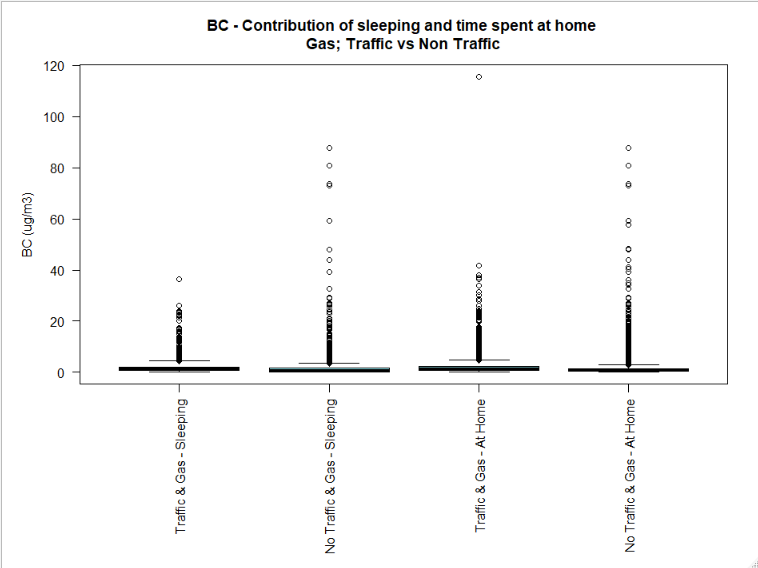 | 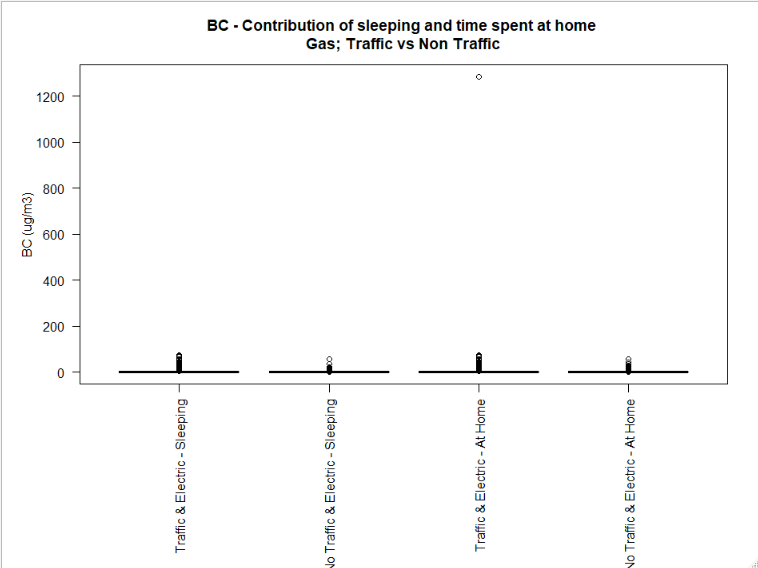 |
| --- | --- |

Figure S5: BC personal exposure concentrations (5-minute time average) during sleeping, and time spent at home, in houses located either near (dark colour) or away (light colour) from busy roads, using either gas (cadet blue/light blue) or electric (dark pink/light pink) stove. The pollutant measurement distributions are non-normal (see main text).

| 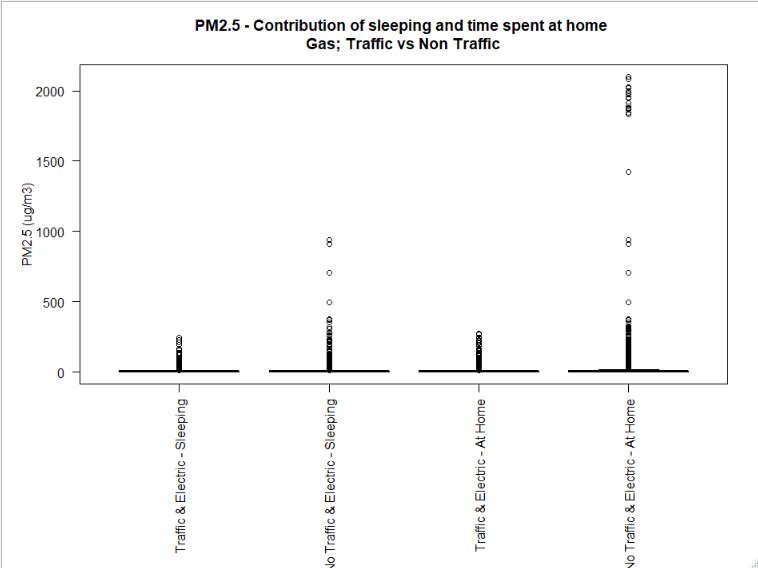 | 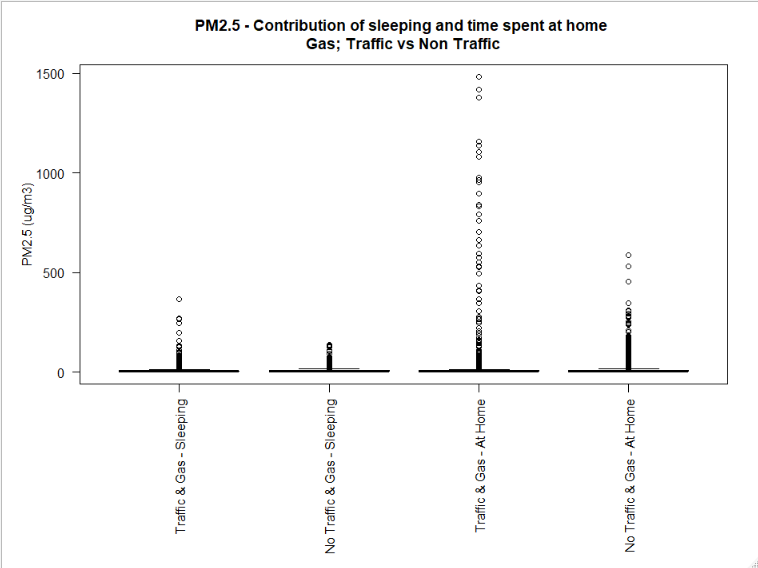 |
| --- | --- |

Figure S6: PM_2.5_ personal exposure concentrations (5-minute time average) during sleeping, and time spent at home, in houses located either near (dark colour) or away (light colour) from busy roads, using either gas (cadet blue/light blue) or electric (dark pink/light pink) stove. The pollutant measurement distributions are non-normal (see main text).
